# How to update a living systematic review and keep it alive during a pandemic: a practical guide

**DOI:** 10.1186/s13643-023-02325-y

**Published:** 2023-09-02

**Authors:** Leonie Heron, Diana Buitrago-Garcia, Aziz Mert Ipekci, Rico Baumann, Hira Imeri, Georgia Salanti, Michel Jacques Counotte, Nicola Low

**Affiliations:** 1 Institute of Social and Preventive Medicine, University of Bern, Bern, Switzerland; 2 Graduate School of Health Sciences, University of Bern, Bern, Switzerland; 3 Jacobs Center for Productive Youth Development, University of Zurich, Zürich, Switzerland; 4 Wageningen Bioveterinary Research, Wageningen University & Research, Lelystad, The Netherlands

**Keywords:** Covid-19, Epidemiology, Public health, Research design

## Abstract

**Background:**

The covid-19 pandemic has highlighted the role of living systematic reviews. The speed of evidence generated during the covid-19 pandemic accentuated the challenges of managing high volumes of research literature.

**Methods:**

In this article, we summarise the characteristics of ongoing living systematic reviews on covid-19, and we follow a life cycle approach to describe key steps in a living systematic review.

**Results:**

We identified 97 living systematic reviews on covid-19, published up to 7th November 2022, which focused mostly on the effects of pharmacological interventions (*n* = 46, 47%) or the prevalence of associated conditions or risk factors (*n* = 30, 31%). The scopes of several reviews overlapped considerably. Most living systematic reviews included both observational and randomised study designs (*n* = 45, 46%). Only one-third of the reviews has been updated at least once (*n* = 34, 35%). We address practical aspects of living systematic reviews including how to judge whether to start a living systematic review, methods for study identification and selection, data extraction and evaluation, and give recommendations at each step, drawing from our own experience. We also discuss when it is time to stop and how to publish updates.

**Conclusions:**

Methods to improve the efficiency of searching, study selection, and data extraction using machine learning technologies are being developed, their performance and applicability, particularly for reviews based on observational study designs should improve, and ways of publishing living systematic reviews and their updates will continue to evolve. Finally, knowing when to end a living systematic review is as important as knowing when to start.

**Supplementary Information:**

The online version contains supplementary material available at 10.1186/s13643-023-02325-y.

## Background

A living systematic review is a systematic review, which is ‘continually updated, incorporating relevant new evidence as it becomes available’ [1]. Researchers are advised to take a living approach when the topic is a priority for decision-making, new evidence is emerging and changing quickly, and certainty in the existing evidence is low [1, 2]. The pandemic of coronavirus disease 2019 (covid-19), caused by the severe acute respiratory syndrome coronavirus 2 (SARS-CoV-2), fulfils these conditions in general, and many living systematic reviews addressing questions about SARS-CoV-2 and covid-19 were published during the pandemic [3–10].

**Fig. 1:**
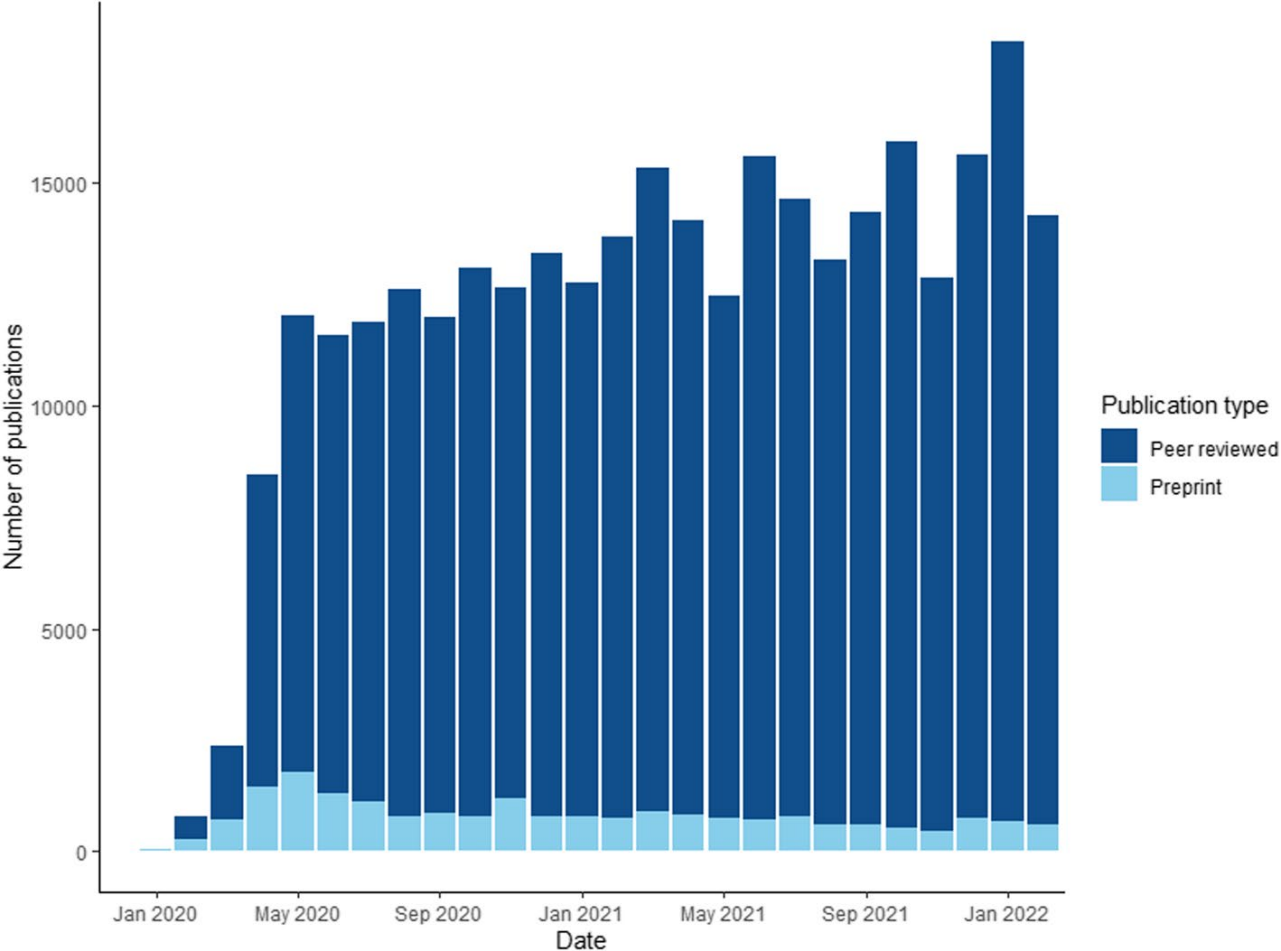
Monthly new records on SARS-CoV-2 or covid-19 from January 2020 to February 2022. Number of new records from five electronic databases (PubMed, Embase, PsychINFO, bioRxiv, and medRxiv)

The speed and sustained accumulation of published research about SARS-CoV-2 and covid-19 since the beginning of 2020 are unprecedented (Fig. 1). By 28 February 2022, more than 314,000 peer-reviewed articles and preprints had been published in five electronic literature databases [11]. After a rapid early surge, around 14,500 articles on SARS-CoV-2 and covid-19 have been published every month (mean publications from January 2021 until February 2022) [11]. Types of publication have changed over time [12], and the evidence itself is changing, for example, as new viral variants arise and new vaccines and treatments are developed.

The evolving evidence on covid-19 is being accompanied by changes in living systematic reviews, which were originally defined as an approach to updating an existing systematic review, not a methodology in itself [1]. Now, many authors describe their review as a living systematic review from the outset [3–10]. In these living systematic reviews, distinctions between approaches recommended for rapid reviews [13] and standard systematic reviews [1], which living systematic reviews are supposed to follow, are also becoming blurred. Rapid review methods include processes to speed up production, such as data extraction by a single reviewer, or limits on search dates or languages, even though some of these practices are judged to increase the risk of bias in systematic reviews [13]. Reviewers who have used these methods have described their study designs as a ‘living rapid review’ on the effectiveness of face masks [14], a ‘rapid living systematic review’ of rehabilitation for covid-19 patients [15], or just a ‘living systematic review’ of asymptomatic SARS-CoV-2 infection [10].

Guidance about methods for several aspects of the conduct, reporting, and publication of living systematic reviews is available [1, 16–19] or in development [20, 21]. The covid-19 pandemic has highlighted not only the methodological considerations when conducting living systematic reviews [19] but also the practical challenges of sustaining a workflow. These challenges include needs for the urgent decision-making [22]; managing high volumes of research, especially on observational study designs; and judging whether to start a living systematic review, when it is time to stop, and how to publish updates [23]. To address these practical aspects of living systematic reviews, we use our own experience of doing a living systematic review on asymptomatic SARS-CoV-2 infections as a case study [24]. We also draw on covid-19-related living systematic reviews on changes in mental health in the general population, SARS-CoV-2 diagnostics, epidemiology of covid-19 in pregnancy, and effectiveness of treatments and vaccines, which cover a variety of methods used to conduct and manage reviews [3–10]. In this article, we summarise the characteristics of ongoing living systematic reviews on covid-19; we follow a life cycle approach to describe key steps in a living systematic review and give recommendations at each step.

## The state of covid-19 evidence and living systematic reviews

To summarise the status of living systematic reviews about SARS-CoV-2 and covid-19, we searched titles of records in the World Health Organization COVID-19 Database [25] using the search term ‘living systematic review’ on 7th November 2022. We did not search for reviews hosted only on websites (Additional file 1). Of 861 hits, we found 97 unique studies described by the authors as living systematic reviews on covid-19 (Table 1 and Additional file 2). These living systematic reviews mainly addressed questions about the effects of pharmacological interventions (therapies and vaccines) (*n* = 46, 47%) and the prevalence of SARS-CoV-2 or covid-19-associated conditions or risk factors (*n* = 30, 31%). Twenty-eight (29%) living systematic reviews included only randomised controlled trials, 22 (23%) reviewed only observational studies, and 45 (46%) reviewed both observational and randomised controlled trials. There were considerable overlap in the scopes of some studies. For example, four reviews focused on the diagnostic accuracy of rapid antigen tests, four on the effectiveness of vaccines, three on long-term symptoms of covid-19, and two on transmission of SARS-CoV-2 from mother to child. The studied populations were mainly people with suspected or diagnosed covid-19 or long COVID (*n* = 61, 63%). They were mostly groups of any ages (*n* = 61, 63%), which included studies on hospital patients (*n* = 23). Studies on adults only (*n* = 34, 35%) included hospital patients (*n* = 13) and healthcare workers (*n* = 5). Most living systematic reviews had published a protocol before the first publication of the review (*n* = 84, 87%), and most had at least one version published in a peer-reviewed journal (*n* = 95, 98%).

**Table 1:** Characteristics of living systematic reviews on covid-19, 1 January 2020 to 7 November 2022

Total	Living systematic reviews *n* (%)
	97 (100)
Research area^*^	
Pharmacological interventions	46 (47)
Prevalence of conditions or risk factors	30 (31)
Diagnostic test accuracy	10 (10)
Health and social care delivery	6 (6)
Nonpharmacological interventions	4 (4)
Prognosis	2 (2)
Research on research	2 (2)
Aetiology	1 (1)
Economic impact	1 (1)
Study design of eligible studies	
Observational and randomised study designs	45 (46)
Randomised controlled trials only	28 (29)
Observational study designs only	22 (23)
Economic evaluation	1 (1)
Guidelines and recommendations	1 (1)
Covid-19 infection status of study population	
Suspected or diagnosed covid-19 or long COVID	61 (63)
Susceptible to covid-19	32 (33)
Mixed group	4 (4)
Age and subgroups of study population	
Any ages	61 (63)
Hospital patients (*n* = 23)	
Cancer survivors (*n* = 1)	
Patients and healthcare workers (*n* = 1)	
Pregnant or recently pregnant women and their children (*n* = 1)	
Students and staff in schools (*n* = 1)	
Adults only (≥ 18 years)	
Hospital patients (*n* = 13)	34 (35)
Healthcare workers (*n* = 5)	
Pregnant and recently pregnant women (*n* = 3)	
People in living in long-term care facilities (*n* = 2)	
Children only (< 18 years)	
Infants born to mothers with confirmed covid-19 (*n* = 1)	2 (2)
Protocol published before first publication	84 (87)
At least one version published in peer-reviewed journal	95 (98)
Any update† available, by date of first publication	34 (35)
January-June 2020 (*n* = 9)	7
July-December 2020 (*n* = 28)	15
January-June 2021 (*n* = 22)	7
July-December 2021 (*n* = 15)	6
January-June 2022 (*n* = 18)	3
July-November 2022 (*n* = 5)	2

^*^Some studies focused on more than one research area. †Published as a preprint, journal publication, or on a study website

Updating with new evidence is a core principle of living systematic reviews [1]. From January to June 2020, 7/9 (78%) living systematic reviews had been updated at least once. Of living systematic reviews published from January to June 2021 (12 to 18 months from the date of the search), only 7/22 (32%) had been updated at least once, which might reflect the large workload associated with a living systematic review. No authors clearly stated that their living systematic review had ended.

The workload for living systematic reviews depends on the review question, the eligible study designs, and the amount of underlying evidence. Review questions that do not restrict their search by study design or rely entirely on observational study designs, such as prevalence or aetiology studies, require more work at the early stages of the review than questions about interventions. One reason is that randomised trials are tagged in databases such as PubMed, there are validated search filters to find them, and guidelines that ensure that important items are reported. Indexing and reporting of observational study designs are less consistent, and authors may use different terms to describe the same approach [26]. A second reason is that the search terms for questions about topics such as prevalence and aetiology tend to be less specific than those for interventions, generating more hits to be screened and from which to extract data.

## Establishing and updating a living systematic review

**Fig. 2:**
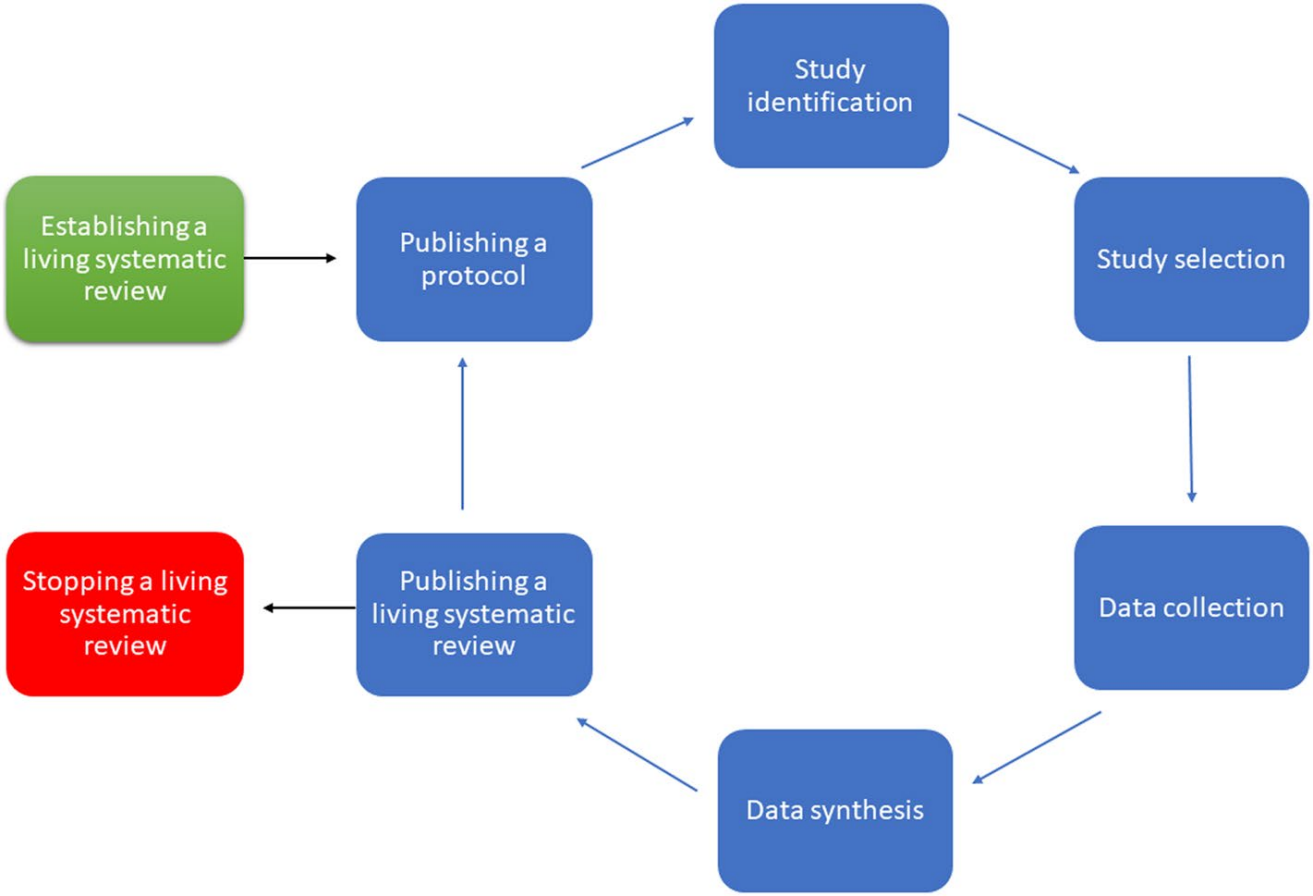
Life cycle of a living systematic review

We summarise the steps in the life cycle of a living systematic review in Fig. 2. In Table 2, we summarise the methods that we used in our own living systematic review [10]. Frequently overlooked is the need to be realistic about the time needed to start and update the living systematic review and plan as many steps as possible in advance, taking into consideration that numbers of records to screen might continue to increase quickly, as has happened with covid-19 literature. Given the commitment required and the scale of the workload, reviewers should make sure that their living systematic review question has not already been addressed, or is being addressed, by searching the published literature, systematic review registries, such as the PROSPERO international register of systematic reviews [3–5, 9], or the Open Science Framework (OSF) [8, 10].

**Table 2:** Summary of a living systematic review of asymptomatic and presymptomatic SARS-CoV-2 infection

*Protocol*: First published 1 April 2020, last updated 13 July 2022 [27]
*Versions*: (1) 29 April 2020 (preprint), (2) 24 May 2020 (preprint), (3) 28 July 2020 (preprint), (4) 22 Sept 2020 (peer-reviewed publication), (5) 30 Jan 2022 (preprint), (6) 26 May 2022 (peer-reviewed publication)
*Research questions*: (1) Among people who become infected with SARS-CoV-2, what proportion does not experience symptoms at all during their infection? (2) What is the infectiousness of people with asymptomatic and presymptomatic, compared with symptomatic SARS-CoV-2 infection? (3) What proportion of SARS-CoV-2 transmission is accounted for by people who are either asymptomatic throughout infection or presymptomatic?
*Inclusion criteria*: Studies of people with SARS-CoV-2 diagnosed by RT-PCR that documented follow-up and symptom status at the beginning and end of follow-up or investigated the contribution to SARS-CoV-2 transmission of asymptomatic or presymptomatic infection
*Exclusion criteria*: Case series restricted to people already diagnosed; studies that did not report the number of people tested for SARS-CoV-2, from whom the study population was derived; case reports; contact investigations of single individuals or families; and any study without sufficient follow-up
*Articles screened and included in review*: The searches for studies about asymptomatic or presymptomatic SARS-CoV-2, on 25 March, 20 April, and 10 June 2020 and 2 February and 6 July 2021 resulted in 89, 230, 688, 4213, and 3018 records for screening, respectively. The latest version included 146 studies in total

### Setting up and managing a review team

The core team should coordinate the tasks of the review team, which includes anticipating and managing changes in workload, workflow, and team composition. At least one core team member should have sufficient programming skills to automate steps in the workflow where possible. In our case study, a core review team of seven became overwhelmed when the number of new hits to be screened increased (Table 2). Growth in the team of researchers can be seen when following updates of other living systematic reviews [8]. Crowdsourcing is a valuable tool for large reviews [17] and can be mutually beneficial for the volunteers [28], but the core team must weigh up the time spent training volunteers against the time saved. Some review teams have anticipated the workload and used crowdsourcing from the outset, increasing the size of the team to more than 100 volunteers to help with screening and data extraction [3], and other reviews mention the use of volunteers [4, 7]. We recruited twenty volunteers from April 2021, through the core team’s networks. All volunteers had previous experience with systematic reviews and agreed to spend at least 3 h per month working on eligibility assessment, data extraction, and/or risk-of-bias assessment. The core team provided online guidance materials (Additional file 3), individual feedback, and automated tools. Training for members of the crowd (Additional file 3) reduced potential disagreements in screening, extra work for the core team, and delays in the living systematic review.

Defining eligibility criteria for authorship is an essential task for the core team at the start of the review process, who should agree the policy in advance with the review team, including crowdsourced members. In our review, levels of contribution and availability changed during and between updates. Team members who fulfilled the criteria for authorship were co-authors of the relevant publication. We created lists of contributorship [29] in which people whose contributions no longer fulfilled criteria for authorship had their contributions acknowledged separately.

### Publishing a protocol

A protocol for a living systematic review is also a living document, which should reduce potential biases and avoid posthoc decisions [1, 18, 30]. Publishing a protocol on PROSPERO [3–5, 9, 15], on a preprint server, or public repositories like the OSF [8, 10] allows rapid sharing and updating of protocols. In a living systematic review, the review questions and scope and types of evidence included might evolve over time, so authors should document and justify changes to the protocol before starting an update, including decisions about the frequency of updating and about stopping the review. If protocol changes are needed, authors should note that the scope of a living systematic review can only become narrower over time without having to make changes to the original search strategy. Over the seven versions of the protocol for our review of asymptomatic SARS-CoV-2, the scope has narrowed over time [27]. In the first version, there were few publications, and we included study populations in any setting. After our third version, we reduced the number of studies for data extraction by excluding small studies reporting on single family contact investigations and studies of hospitalised people, who were more likely to be symptomatic. 

### Study identification

Automatic alerts from bibliographic databases can notify researchers when new records are available [17]. For complex reviews, researchers with sufficient programming skills can set up automatic scripts to regularly search and collect results from search databases using programming languages, either with an application programme interface (API) (a software intermediary that communicates with websites from a third-party application) or by ‘web scraping’. Database aggregators are convenient, single sources for a topic of interest; information scientists develop, refine, automate, and update search strings in different electronic sources and de-duplicate the records. Database aggregators for covid-19 literature include the World Health Organization COVID-19 Database and the Cochrane COVID-19 Trials Register (https://covid-19.cochrane.org/). We used the COAP living evidence database, a database aggregator [11], which we ran from March 2020 to March 2022 [10]. We scheduled an automated R script [31] to search COAP weekly, using the task scheduler, cron. Each week, the automatic search uploaded 100–200 new records from the COAP database for our living systematic review. We searched preprint servers and included preprints if they fulfilled eligibility criteria. In each update, we checked the status of preprints to see if they had been published in peer-reviewed journals and re-extracted data if the content had changed.

Electronic online databases to save and manage records support a secure and efficient workflow. Living systematic reviewers are using tools such as Evidence for Policy and Practice Information (EPPI)-reviewer [32], Covidence [6, 33, 34], or Microsoft Excel to organise records. New records in our review (Table 2) are saved in a Research Electronic Data Capture (REDCap) database [35], a flexible and secure online system. A copy of the data is stored in a collaborative software repository [36].

### Study selection

**Fig. 3:**
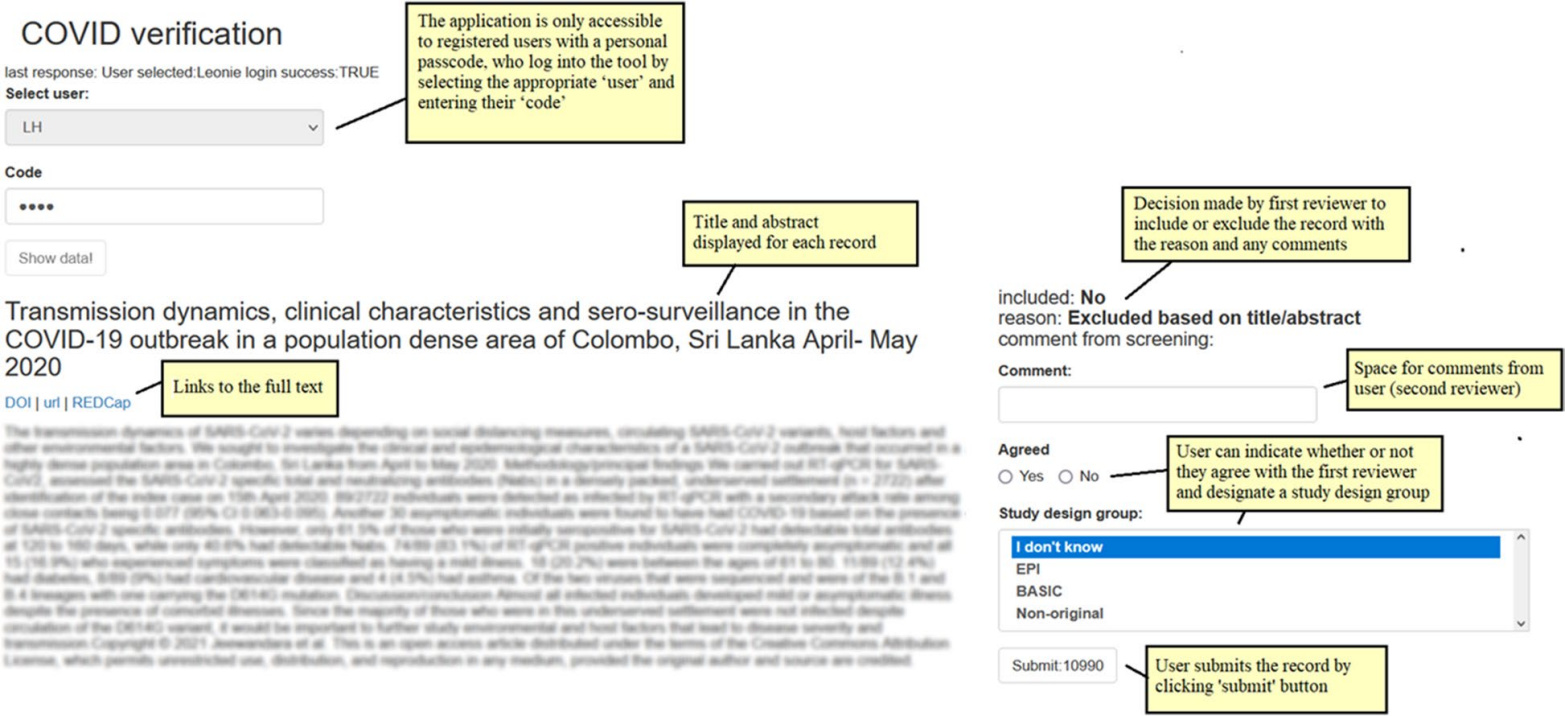
Annotated screenshot of the R Shiny application used for the selection process. The RShiny application was developed for the reviewing team of the living systematic review on asymptomatic SARS-CoV-2 infections to screen and verify articles

Several software tools offer fast and user-friendly platforms to facilitate screening records [33]. Living systematic reviews on covid-19 has used REDCap surveys [3, 35], EPPI-reviewer [8, 32], and Covidence [6, 34]. The tools support multiple users, allocate tasks, record decisions, and produce automatic reports [33]. The open-source R package revtools [37] support the screening of titles and abstracts and deduplication. When specific features are desired, or if software licences are unaffordable, building a custom application using open-source software might be more suitable. We constructed password-protected R Shiny applications to support the selection process (Fig. 3) [10]. The core team allocates records to the reviewing team via REDCap [35]. The applications included features to allow the team to train a machine learning algorithm (see below).

Semiautomated machine learning tools for the selection process can reduce the volume of studies that needs to be screened manually [38]. However, the tools may not perform as well for observational studies as for RCTs, for which accepted reporting guidelines and terminology facilitate reliable identification of reports [38]. Wynants et al. built a custom classification model to speed up the selection process in their living systematic review of prognostic models for covid-19 [8]. They used the initial set of records that they screened to train an algorithm to recognise patterns in text to identify studies that are very unlikely to be relevant and automatically exclude them. For reporting the results of searching, selection, and inclusion, a specific flow chart for living systematic reviews allows a logical way of updating [39].

### Data collection

Web applications may help to streamline manual data extraction by reviewers who are extracting information independently or verifying the information extracted by another reviewer, including using the use of online forms [4, 7], REDCap surveys [3], or standardised prespecified extraction forms [6, 8, 9, 14]. None of these living systematic reviews mentioned the use of automated tools for data collection. We used RShiny applications to facilitate both steps and save decisions in REDCap [10, 35]. While machine learning tools for data extraction exist, very few are publicly available [40]. The tools face challenges with variations in wording, missing information, and adaptability to a subject area on which the tool was not developed [40].

### Data synthesis

Manual checks on included studies are still needed before starting data synthesis, especially when a large crowd has contributed to selection of studies and extraction of data and rapid processes have been put in place. Routine checks include making sure that data have not been included from a preprint and a published version of the same study. There are many statistical software packages for conducting quantitative data synthesis for living systematic reviews, including Stata [41] and R [4–6, 9, 10, 31]. The use of an API to communicate between an online database and the statistical software allows reviewers to import the latest data and update the analysis when new data are available. Reviewers can generate tables and figures automatically using statistical software (e.g. R Markdown [31], Stata [41]).

There are issues associated with repeated updating of statistical analysis, which are particularly relevant in living systematic reviews. With each update, the analysis of data from RCTs or comparative effectiveness studies is more likely to generate a false statistically significant result [16]. Even when the aim of the living systematic review is to support a decision, e.g. to decide which intervention is more effective, statistical significance is rarely the only criterion guiding this decision. However, reviewers can employ methods that control the type I error if they want [16, 42].

A substantial proportion of living systematic reviews rely on observational studies (Table 1), in which levels of between heterogeneity are often high [43], and for which meta-analysis might not be appropriate. Although several living systematic reviews on covid-19 have conducted meta-analyses [4, 6], some did not, owing to high heterogeneity in included studies [10, 14]. In our living systematic review (Table 2), between-study heterogeneity has increased with each update, contrary to our expectation, and we could not explain most of the heterogeneity [10]. In the fifth and sixth versions of the review, we did not produce a summary estimate for the proportion of asymptomatic SARS-CoV-2 infection. Instead, we reported an interquartile range for the results from included studies and estimated a prediction interval [44] to show the range of values in a future hypothetical study.

### Publishing a living systematic review

Living systematic reviews should be published in a way that explicitly cross-references different versions of the report as updates of the same review [20]. These links are needed to make sure that readers have access to the most recent update, and that different versions of a living systematic review are not mistaken for redundant publications. Reviewers should consider contacting the editors of their target journal to find out whether they can submit a living systematic review and to find out how the journal handles updates. Editors of online publications, print publications, and preprint servers use different methods and apply different rules about what they consider a ‘version of record’ [45], which refers to the version of an article that is considered final and is identified online with a digital object identifier (DOI). For living systematic reviews, the version of record is not defined consistently across journals [22]. Different publishers apply different rules to determine whether an update receives the same DOI as a previous version or a new DOI. This decision can depend on whether the journal editors consider and update as minor or major. The BMJ assigns the same DOI to all versions of a living systematic review and adds a ‘reader’s note’ to the abstract, signalling the update number and how to find earlier updates [8]. Cochrane reviews have indexed updates for many years and assign a DOI that incorporates the same review number for all updates and includes an extension with the update number [4]. Newer online publishers, such as F1000, use the same principle as the Cochrane library and also include the version number in all article titles [40]. The Public Library of Science (PLOS) publishes minor updates as online comments to the earlier version and assigns a new DOI if the editors consider it a major update [10]. The *Annals of Internal Medicine* uses a similar approach, with minor updates published as letters [14, 46]. Preprints are not considered a version of record. The medRxiv server allows updates to a living systematic review to be uploaded under the same DOI (available in the history of the article) until the review is published in a peer-reviewed publication [24, 47]. After that, only a major update can be uploaded, and that version receives a new DOI, again, until published. In our case study (Table 2), we have published both preprints [24, 47] and peer-reviewed articles [10, 48].

Transparency is important when sharing results of living systematic reviews. Living systematic review and living guidelines teams who maintain dedicated websites can display updated results as soon as they are incorporated and include links to articles, protocols, and datasets using FAIR principles (findability, accessibility, interoperability, and reuse of digital assets) [3–6, 8, 10, 22].

### Stopping a living systematic review

An important feature of a living systematic review is knowing when to stop, and the criteria for stopping should be part of the review protocol, updated if necessary. Covid-19 living systematic review teams have reported a predefined point at which they intend to stop: either a specific month [3, 4, 9] or when new evidence is unlikely to emerge [6]. In our review, we stated the following criteria for ending the review: when estimates are stable and unlikely to change or the question is no longer of importance [10]. Alternatively, publishers or available funding may determine the lifetime of a living review. The BMJ has set a duration of 2 years for a living systematic review, after which the editors and authors should assess the need for continuation [23].

## Conclusions

The covid-19 pandemic has highlighted the importance of living systematic reviews and living evidence. The volume and speed of evidence generated during the covid-19 pandemic have exceeded expectations since the concept of living systematic reviews was first elaborated. Living systematic reviews have an intense workload, and it is especially important to avoid the research waste of reviews with overlapping scope, such as the 3 reviews on the effectiveness of vaccines that we found in our review of living systematic reviews. Living systematic reviews should be updated as new evidence becomes available, but several studies described as living systematic reviews on covid-19 have not been updated since publication of the first version (*n* = 63, 65%) [3–5, 9]. This highlights the difficulty of keeping a living systematic review alive. In this article, we have summarised the processes in a living systematic review as a life cycle (Fig. 2), described practical considerations at each step, and made recommendations (Table 3), drawing on a case study from our own experiences (Table 2). Methods to improve the efficiency of searching, study selection, and data extraction using machine learning technologies are being developed; their performance and applicability, particularly for reviews based on observational study designs, should improve; and ways of publishing living systematic reviews and their updates will continue to evolve. Finally, knowing when to end a living systematic review is as important as knowing when to start.

**Table 3:** Recommendations for conducting a living systematic review, by stage of the review life cycle

*Establishing a living systematic review*	• Initiate a living systematic review only when the topic is a priority for decision-making and the evidence is uncertain and/or is changing quickly • Estimate the time needed to conduct and update the living systematic review realistically • Anticipate increasing numbers of records to screen with successive updates
*Publishing a protocol*	• Publish and register a protocol before starting the living systematic review • Explicitly state the conditions for ending the living systematic review • Document changes in a new protocol version before starting the next update
*Setting up and managing the review team*	• The team should have appropriate subject area and methodological and technical expertise • Consider crowdsourcing volunteers to help with time-intensive tasks that require less content expertise
*Study identification*	• Automate searches, e.g. by using statistical software and application programme interface (APIs) to communicate with online database aggregators • Store and manage identified records in a secure, online electronic database
*Study selection and data collection*	• Use software tools (e.g. Covidence, REDCap surveys) to organise and facilitate screening records • Combine screening of titles and abstracts and full texts into one step • Use text classifiers to automatically exclude ineligible articles, if appropriate
*Data synthesis*	• Synthesise the data using statistical software that can connect to an electronic database • Create reproducible documents, tables, and/or figures to quickly update results when new studies are included • Consider statistical issues associated with multiple updates
*Publishing the results*	• Publish updates first as preprints and then as open-access, peer-reviewed publications • Choose an appropriate platform (i.e. journal, preprint server, or website) that makes the version of the review clear • Consider a living systematic review website for sharing updates
*Ending a living systematic review*	• Decide on and state criteria for ending the living systematic review in the review protocol

### Supplementary Information

**Additional file 1. **Summary of methods used in a review of living systematic reviews on covid-19.

**Additional file 2. **Living systematic reviews on covid-19 identified in the World Health Organization COVID-19 Database from January 2020 to February 2022, ordered alphabetically by first author.

**Additional file 3. **Training materials for voluntary team members in the living systematic review.

## Data Availability

All data generated or analysed during this study are included in this published article and its supplementary information files.

## Abbreviations

API: Application programme interface
Covid-19: Coronavirus disease 2019
DOI: Digital object identifier
EPPI: Evidence for policy and practice information
FAIR: Findability, accessibility, interoperability, and reuse of digital assets
OSF: Open Science Framework
PLOS: Public Library of Science
REDCap: Research Electronic Data Capture
SARS-CoV-2: Severe acute respiratory syndrome coronavirus 2
