# Supplementary material for: How to update a living systematic review and keep it alive during a pandemic: a practical guide
Source: Syst Rev. 2023 Sep 2;12:156. doi: 10.1186/s13643-023-02325-y (PMC10474670; doi:10.1186/s13643-023-02325-y)
Supplement: Supplementary file 1 — Additional file 1. Summary of methods used in a review of living systematic reviews on covid-19. [file 13643_2023_2325_MOESM1_ESM.docx]

Additional file 1. Summary of methods used in a review of living systematic reviews on covid-19

**Information sources and search**

We searched the World Health Organization COVID-19 Database (<https://search.bvsalud.org/global-literature-on-novel-coronavirus-2019-ncov/>) on 7^th^ November 2022 using the search term ‘living systematic review’. All literature in the database is on coronavirus disease 2019.

**Eligibility criteria**

We included studies which authors described as a living systematic review anywhere in the title, abstract, full-text, or protocol. There were no language restrictions, and we excluded all other study designs.

**Study selection and data extraction**

One reviewer (Rico Baumann, Diana Buitrago-Garcia or Leonie Heron) screened the records and decided on inclusion, asking a second reviewer (Rico Baumann, Diana Buitrago-Garcia or Leonie Heron) to verify the decision in cases of uncertainty. We identified the number of unique living systematic reviews and extracted data from the studies.

Two reviewers (Rico Baumann, Diana Buitrago-Garcia or Leonie Heron) separately extracted data from the studies. Disagreements were resolved by Nicola Low. The extracted variables were study authors, DOI, journal name, study title, date first published, format of publication (journal, preprint, or other), availability and location of protocol, number of study updates, date of last update, review topic, area of research (aetiology; diagnostic test accuracy; economic impact; health and social care delivery; nonpharmacological interventions; pharmacological interventions; prevalence of conditions or risk factors; prognosis; or research on research), study population and types of eligible studies in review (observational studies, randomised controlled trials, both observational studies and randomised controlled trials, economic evaluations, or guidelines).

**Synthesis of the evidence**

We describe the key characteristics of the identified living systematic reviews in a summary table in the main text. A condensed table of the individual living systematic reviews is provided in supplementary online material 1 and a full version available on the Open Science Framework (https://osf.io/6nr7q/).
