# Supplementary material for: How to update a living systematic review and keep it alive during a pandemic: a practical guide
Source: Syst Rev. 2023 Sep 2;12:156. doi: 10.1186/s13643-023-02325-y (PMC10474670; doi:10.1186/s13643-023-02325-y)
Supplement: Supplementary file 2 — Additional file 2. Living systematic reviews on covid-19 identified in the World Health Organization COVID-19 Database from January 2020 to February 2022, ordered alphabetically by first author. [file 13643_2023_2325_MOESM2_ESM.docx]

Additional file 2. Living systematic reviews on covid-19 identified in the World Health Organization COVID-19 Database from January 2020 to February 2022, ordered alphabetically by first author

| **First author** | **Date of first publication** | **DOI** | **Protocol available** | **Updates available, n** | **Research area** | **Covid-19 infection status of study population** | **Study design of eligible studies*** |
| --- | --- | --- | --- | --- | --- | --- | --- |
| Allotey et al. | 01/09/2020 | 10.1136/bmj.m3320 | YES | 2 | Prevalence | Any covid-19 infection status | 3 |
| Allotey et al. | 16/03/2022 | 10.1136/bmj-2021-067696 | YES | 0 | Prevalence | Susceptible to covid-19 | 1 |
| Amorim Dos Santos et al. | 11/09/2020 | 10.1177%2F0022034520957289 | YES | 1 | Prevalence | Suspected or diagnosed covid-19 or long COVID | 1 |
| Ang et al. | 20/06/2022 | 10.3389/fphar.2022.906764 | YES | 0 | Intervention | Suspected or diagnosed covid-19 or long COVID | 2 |
| Arevalo-Rodriguez et al. | 01/05/2020 | 10.1371/journal.pone.0242958 | YES | 3 | Diagnostic test accuracy | Susceptible to covid-19 | 1 |
| Asiimwe et al. | 07/06/2021 | 10.1111/bcp.14927 | YES | 0 | Intervention | Suspected or diagnosed covid-19 or long COVID | 3 |
| Au et al. | 31/05/2022 | 10.1136/bmj-2022-069989 | YES | 0 | Intervention | Susceptible to covid-19 | 3 |
| Bach-Mortensen et al. | 07/10/2021 | 10.1038/s43587-021-00106-7 | YES | 0 | Health and social care delivery | Susceptible to covid-19 | 1 |
| Baladia et al. | 28/07/2020 | 10.5867/medwave.2020.06.7978 | YES | 0 | Intervention | Suspected or diagnosed covid-19 or long COVID | 3 |
| Bartoszko et al. | 26/04/2021 | 10.1136/bmj.n949 | YES | 0 | Intervention | Susceptible to covid-19 | 2 |
| Batista et al. | 06/06/2022 | https://dx.doi.org/10.36416/1806-3756/e20220041 | YES | 0 | Intervention | Suspected or diagnosed covid-19 or long COVID | 2 |
| Boet et al. | 30/09/2022 | 10.28920/dhm52.2.126-135 | YES | 1 | Intervention | Suspected or diagnosed covid-19 or long COVID | 3 |
| Bonardi et al. | 11/03/2022 | 10.1177/07067437211070648 | YES | 0 | Intervention | Susceptible to covid-19 | 2 |
| Bruemmer et al. | 12/08/2021 | 10.1371/journal.pmed.1003735 | YES | 1 | Diagnostic test accuracy | Suspected or diagnosed covid-19 or long COVID | 3 |
| Buitrago-Garcia et al. | 22/09/2020 | 10.1371/journal.pmed.1003346 | YES | 1 | Prevalence | Suspected or diagnosed covid-19 or long COVID | 1 |
| Bwire et al. | 22/10/2020 | 10.1002/jmv.26622 | YES | 0 | Prevalence | Susceptible to covid-19 | 3 |
| Centeno-Tablante et al. | 28/08/2020 | 10.1111/nyas.14477 | YES | 0 | Prevalence | Suspected or diagnosed covid-19 or long COVID | 3 |
| Ceravolo et al. | 22/04/2020 | 10.23736/s1973-9087.20.06501-6 | YES | 2 | Intervention | Suspected or diagnosed covid-19 or long COVID | 3 |
| Cevik et al. | 28/07/2020 | 10.1016/s2666-5247(20)30172-5 | YES | 0 | Aetiology | Suspected or diagnosed covid-19 or long COVID | 3 |
| Chai et al. | 14/05/2020 | 10.1002/14651858.cd013600.pub2 | YES | 3 | Intervention | Suspected or diagnosed covid-19 or long COVID | 3 |
| Cruciani et al. | 01/02/2021 | 10.1701/3565.35458 | NO | 0 | Intervention | Suspected or diagnosed covid-19 or long COVID | 2 |
| Décary et al. | 18/06/2021 | 10.1101/2021.11.17.21266404 | YES | 1 | Health and social care delivery | Suspected or diagnosed covid-19 or long COVID | 3 |
| Deeks et al. | 25/06/2020 | 10.1002/14651858.cd013652 | NO | 0 | Diagnostic test accuracy | Any covid-19 infection status | 1 |
| Dinnes et al. | 26/08/2020 | 10.1002/14651858.cd013705 | YES | 1 | Diagnostic test accuracy | Any covid-19 infection status | 1 |
| Dong et al. | 01/09/2021 | 10.1016/j.jad.2021.05.060 | YES | 0 | Prevalence | Suspected or diagnosed covid-19 or long COVID | 1 |
| Dzinamarira et al. | 12/04/2022 | 10.1016/j.shaw.2022.04.001 | YES | 0 | Prevalence | Susceptible to covid-19 | 3 |
| Ebrahimzadeh et al. | 30/09/2020 | 10.1002/14651858.CD013639.pub4 | YES | 3 | Diagnostic test accuracy | Suspected or diagnosed covid-19 or long COVID | 3 |
| Elvidge et al. | 16/02/2022 | 10.1016/j.jval.2022.01.001 | YES | 0 | Intervention and diagnostic test accuracy | Suspected or diagnosed covid-19 or long COVID | 4 |
| Fragkou et al. | 24/05/2022 | 10.1177/17562864221099472 | YES | 0 | Intervention | Suspected or diagnosed covid-19 or long COVID | 1 |
| Ghosn et al. | 18/03/2021 | 10.1002/14651858.CD013881 | YES | 0 | Intervention | Suspected or diagnosed covid-19 or long COVID | 2 |
| Gómez-Ochoa et al. | 01/09/2020 | 10.1093/aje/kwaa191 | NO | 0 | Prevalence | Susceptible to covid-19 | 1 |
| Griesel et al. | 09/03/2022 | 10.1002/14651858.cd015125 | YES | 0 | Intervention | Suspected or diagnosed covid-19 or long COVID | 2 |
| Harder et al. | 15/06/2021 | 10.2807%2F1560-7917.ES.2021.26.28.2100563 | YES | 2 | Intervention | Susceptible to covid-19 | 3 |
| Hawthorne et al. | 08/11/2021 | 10.1101/2021.09.20.21263509 | YES | 0 | Diagnostic test accuracy | Susceptible to covid-19 | 1 |
| Hernandez et al. | 18/08/2020 | 10.7326/M20-2496 | YES | 0 | Intervention | Susceptible to covid-19 | 3 |
| Hernández et al. | 24/05/2022 | 10.1038/s41432-022-0253-z | YES | 0 | Intervention | Suspected or diagnosed covid-19 or long COVID | 2 |
| Hoshijima et al. | 11/04/2021 | 10.1101/2021.04.08.21255109 | YES | 0 | Prevalence | Suspected or diagnosed covid-19 or long COVID | 1 |
| Hunter et al. | 04/11/2020 | 10.1101/2020.11.02.20220038 | YES | 0 | Intervention | Suspected or diagnosed covid-19 or long COVID | 2 |
| Hussain et al. | 18/11/2021 | 10.3390%2Fjof7110985 | NO | 0 | Prevalence | Suspected or diagnosed covid-19 or long COVID | 1 |
| Jammu et al. | 26/11/2020 | 10.1007/s00520-020-05908-w | NO | 0 | Health and social care delivery and prevalence | Susceptible to covid-19 | 3 |
| John et al. | 01/05/2020 | 10.12688%2Ff1000research.25522.2 | YES | 1 | Prevalence | Susceptible to covid-19 | 1 |
| Juul et al. | 17/09/2020 | 10.1371/journal.pone.0248132 | YES | 1 | Intervention | Suspected or diagnosed covid-19 or long COVID | 2 |
| Kim et al. | 06/07/2022 | 10.3390/v14071479 | YES | 0 | Diagnostic test accuracy | Suspected or diagnosed covid-19 or long COVID | 3 |
| Kirkham et al. | 31/01/2022 | 10.1016/j.jcyt.2021.12.001 | YES | 1 | Intervention | Suspected or diagnosed covid-19 or long COVID | 3 |
| Knipe et al. | 01/06/2022 | 10.1371/journal.pgph.0000282 | YES | . | Prevalence | Susceptible to covid-19 | 3 |
| Kramer et al. | 13/06/2022 | 10.1002/14651858.CD015209 | YES | 0 | Intervention | Suspected or diagnosed covid-19 or long COVID | 2 |
| Kreuzberger et al. | 02/09/2021 | 10.1002/14651858.cd013825.pub2 | YES | 1 | Intervention | Suspected or diagnosed covid-19 or long COVID | 3 |
| Külper-Schiek et al. | 27/05/2022 | 10.3389/fimmu.2022.940562 | YES | 2 | Intervention | Susceptible to covid-19 | 3 |
| Kwasi Korang et al. | 21/01/2022 | 10.1371/journal.pone.0260733 | YES | 0 | Intervention | Susceptible to covid-19 | 2 |
| Langford et al. | 22/07/2020 | 10.1016/j.cmi.2020.07.016 | YES | NA | Prevalence | Suspected or diagnosed covid-19 or long COVID | 3 |
| Lapitan et al. | 28/04/2021 | 10.47895/amp.v55i2.2875 | NO | 0 | Intervention | Susceptible to covid-19 | 2 |
| Lee et al. | 01/07/2021 | 10.1016/j.amjsurg.2020.11.019 | NO | 0 | Health and social care delivery | Susceptible to covid-19 | 3 |
| Littlewood et al. | 12/10/2022 | 10.1136/ebmental-2022-300530 | YES | . | Intervention | Susceptible to covid-19 | 2 |
| Ma et al. | 14/10/2022 | 10.1101/2022.10.13.22280957 | YES | 2 | Prevalence and diagnostic test accuracy | Susceptible to covid-19 | 1 |
| Mackey et al. | 15/03/2021 | 10.7326/m20-7547 | YES | 1 | Intervention | Suspected or diagnosed covid-19 or long COVID | 3 |
| Mackey et al. | 04/08/2020 | 10.7326/m20-1515 | YES | 9 | Prevalence | Susceptible to covid-19 | 3 |
| Maguire et al. | 02/06/2020 | 10.12688/wellcomeopenres.15933.1 | YES | 1 | Research on research | Suspected or diagnosed covid-19 or long COVID | 2 |
| Melo et al. | 29/06/2021 | 10.1371/journal.pone.0253894 | YES | 0 | Prevalence | Suspected or diagnosed covid-19 or long COVID | 1 |
| Meza et al. | 03/03/2021 | 10.5867/medwave.2021.02.8105 | YES | 0 | Intervention | Suspected or diagnosed covid-19 or long COVID | 2 |
| Michelen et al. | 27/09/2021 | 10.1136/bmjgh-2021-005427 | YES | 1 | Prevalence | Suspected or diagnosed covid-19 or long COVID | 3 |
| Mikolajewska et al. | 18/10/2021 | 10.1002/14651858.cd015045 | YES | 0 | Intervention | Suspected or diagnosed covid-19 or long COVID | 2 |
| Norton et al. | 08/09/2020 | 10.12688/wellcomeopenres.16259.5 | NO | 4 | Research on research | Susceptible to covid-19 | 3 |
| O'Byrne et al. | 22/07/2021 | 10.1002/14651858.cd013876.pub2 | YES | 0 | Intervention | Suspected or diagnosed covid-19 or long COVID | 2 |
| Qiu et al. | 03/09/2020 | 10.1016/j.cmi.2021.01.011 | YES | 3 | Prevalence | Suspected or diagnosed covid-19 or long COVID | 1 |
| Rada et al. | 27/12/2020 | 10.5867/medwave.2020.11.8078 | YES | 0 | Intervention | Suspected or diagnosed covid-19 or long COVID | 3 |
| Reis et al. | 20/09/2022 | 10.1002/14651858.CD015395.pub2 | YES | . | Intervention | Any covid-19 infection status | 2 |
| Reyes Domingo et al. | 06/06/2021 | 10.1101/2021.06.03.21258317 | YES | 1 | Prevalence | Suspected or diagnosed covid-19 or long COVID | 3 |
| Rocha et al. | 17/12/2020 | 10.1590/1516-3180.2020.0421.r2.10092020 | YES | 0 | Intervention | Suspected or diagnosed covid-19 or long COVID | 3 |
| Rodríguez-Gutiérrez et al. | 11/03/2021 | 10.2139/ssrn.3802499 | YES | 0 | Intervention | Suspected or diagnosed covid-19 or long COVID | 2 |
| Salah Eddine Oussama et al. | 21/12/2021 | 10.1101/2021.12.19.21268044 | NO | 0 | Intervention | Suspected or diagnosed covid-19 or long COVID | 3 |
| Salcher-Konrad et al. | 09/06/2020 | 10.1101/2020.06.09.20125237 | YES | 2 | Prevalence | Susceptible to covid-19 | 3 |
| Schlesinger et al. | 28/04/2021 | 10.1007/s00125-021-05458-8 | YES | 0 | Prognosis | Suspected or diagnosed covid-19 or long COVID | 3 |
| Schünemann et al. | 04/08/2020 | 10.7326/m20-2306 | YES | 3 | Intervention | Suspected or diagnosed covid-19 or long COVID | 3 |
| Seo Kim et al. | 01/02/2021 | 10.1002/rmv.2336 | YES | 0 | Intervention | Susceptible to covid-19 | 3 |
| Siemieniuk et al. | 30/07/2020 | 10.1136/bmj.m2980 | YES | 4 | Intervention | Suspected or diagnosed covid-19 or long COVID | 2 |
| Siemieniuk et al. | 23/09/2021 | 10.1136/bmj.n2231 | YES | 0 | Intervention | Suspected or diagnosed covid-19 or long COVID | 2 |
| Silveira et al. | 12/02/2022 | 10.1016/j.archoralbio.2022.105374 | YES | 0 | Prevalence | Suspected or diagnosed covid-19 or long COVID | 1 |
| Sirois et al. | 28/01/2021 | 10.3389/fpsyt.2020.589545 | YES | 0 | Prevalence | Susceptible to covid-19 | 1 |
| Soriano et al. | 10/05/2020 | 10.3390/nu13062060 | NO | 0 | Prevalence | Suspected or diagnosed covid-19 or long COVID | 3 |
| Soto-Cámara et al. | 27/11/2021 | 10.3390/jcm10235578 | YES | 0 | Prevalence | Susceptible to covid-19 | 3 |
| Stamm et al. | 07/07/2020 | 10.1101/2020.06.19.20134767 | NO | 2 | Health and social care delivery | Susceptible to covid-19 | 5 |
| Stroehlein et al. | 24/05/2021 | 10.1002/14651858.CD015043 | YES | 0 | Intervention | Suspected or diagnosed covid-19 or long COVID | 2 |
| Sun et al. | 11/05/2021 | 10.1101/2021.05.10.21256920 | YES | 0 | Prevalence | Susceptible to covid-19 | 3 |
| Thakar et al. | 25/01/2022 | 10.12688/f1000research.55109.2 | YES | 0 | Intervention | Suspected or diagnosed covid-19 or long COVID | 3 |
| Tleyjeh et al. | 05/11/2020 | 10.1016/j.cmi.2020.10.036 | NO | 1 | Intervention | Suspected or diagnosed covid-19 or long COVID | 3 |
| Vasco-Morales et al. | 17/05/2021 | 10.15446/revfacmed.v69n1.90222 | YES | 0 | Prevalence | Suspected or diagnosed covid-19 or long COVID | 1 |
| Verdejo et al. | 14/12/2020 | 10.5867/medwave.2020.11.8073 | YES | 0 | Intervention | Suspected or diagnosed covid-19 or long COVID | 2 |
| Verdugo-Paiva et al. | 15/07/2020 | 10.5867/medwave.2020.06.7966 | YES | 0 | Intervention | Suspected or diagnosed covid-19 or long COVID | 3 |
| Verdugo-Paiva et al. | 09/12/2020 | 10.5867/medwave.2020.11.8080 | YES | 0 | Intervention | Suspected or diagnosed covid-19 or long COVID | 2 |
| Vindrola-Padros et al. | 23/03/2021 | 10.1101/2020.10.07.20208587 | YES | 0 | Health and social care delivery | Suspected or diagnosed covid-19 or long COVID | 3 |
| Wagner et al. | 16/08/2021 | 10.1002/14651858.cd014963 | YES | 0 | Intervention | Suspected or diagnosed covid-19 or long COVID | 2 |
| Webster et al. | 22/07/2021 | 10.1002/14651858.cd013877.pub2 | YES | 1 | Intervention | Suspected or diagnosed covid-19 or long COVID | 2 |
| Wilt et al. | 01/02/2021 | 10.7326/m20-5752 | NO | 5 | Intervention | Suspected or diagnosed covid-19 or long COVID | 2 |
| Wynants et al. | 07/04/2020 | 10.1136/bmj.m1328 | YES | 3 | Prognosis and diagnostic test accuracy | Susceptible to covid-19 | 1 |
| Xu et al. | 19/12/2020 | 10.7189%2Fjogh.10.021104 | YES | 0 | Prevalence | Susceptible to covid-19 | 1 |
| Yang et al. | 06/06/2021 | 10.1111/aogs.14206 | YES | 2 | Prevalence | Susceptible to covid-19 | 3 |
| Zhang et al. | 27/01/2022 | 10.3389%2Ffmed.2021.800492 | YES | 0 | Intervention | Suspected or diagnosed covid-19 or long COVID | 3 |
| *Study design: observational study designs only = 1; randomised controlled trials only = 2; observational and randomised study designs = 3; economic evaluation = 4; guidelines and recommendations = 5.  DOI, digital object identifier; RCT, randomised controlled trial.  A complete version of this table is available online at https://osf.io/6nr7q/ | | | | | | | |
