# Supplementary material for: How to update a living systematic review and keep it alive during a pandemic: a practical guide
Source: Syst Rev. 2023 Sep 2;12:156. doi: 10.1186/s13643-023-02325-y (PMC10474670; doi:10.1186/s13643-023-02325-y)
Supplement: Supplementary file 3 — Additional file 3. Training materials for voluntary team members in the living systematic review. [file 13643_2023_2325_MOESM3_ESM.docx]

Additional file 3. Training materials for voluntary team members in the living systematic review

| **Training material and meetings** | **URL** |
| --- | --- |
| Guidelines for screening studies for question 1 in the living systematic review | <https://www.youtube.com/watch?v=4nQk-li_oTw&list=PLt5MrtgUHf71khpVoJxiOdlDhDvY6yZJK&index=1> |
| Guidelines for screening studies for question 1 in the living systematic review | <https://www.youtube.com/watch?v=PVhWPPG_-ig&list=PLt5MrtgUHf71khpVoJxiOdlDhDvY6yZJK&index=2> |
| Guidelines on how to use software and conduct data extraction in the living systematic review | <https://www.youtube.com/watch?v=Br0YOEp4Qig&list=PLt5MrtgUHf71khpVoJxiOdlDhDvY6yZJK&index=3> |
| Recording of crowd meeting – 22 June 2021 | <https://www.youtube.com/watch?v=nP2bQtsdxYY&list=PLt5MrtgUHf71khpVoJxiOdlDhDvY6yZJK&index=4> |
| Recording of crowd meeting – 14 December 2021 | <https://www.youtube.com/watch?v=JU2V4kWoTMI> |
